# Machine Learning as a Tool in Investigating the Possible Role of Microbiome in Development and Treatment of Cancer

**DOI:** 10.7759/cureus.17415

**Published:** 2021-08-24

**Authors:** Sreehita Hajeebu, Ngonack J Ngembus, Pushyami Satya Bandi, Preetish Kumar Panigrahy, Stacey Heindl

**Affiliations:** 1 Medicine, California Institute of Behavioral Neurosciences & Psychology, Fairfield, USA; 2 Pharmacology, California Institute of Behavioral Neurosciences & Psychology, Fairfield, USA; 3 Neurology, California Institute of Behavioral Neurosciences & Psychology, Fairfield, USA

**Keywords:** gut microbiota, oncogenes and microbiota, gut cancer, machine learning, inflammation, artificial intelligence

## Abstract

In recent times, cancer has become a leading cause of death worldwide, and a need for new therapeutic methods to save lives has become an inevitable necessity. Microbiome and its composition have been a key area of interest among the scientific community. Microbiota appears to hold the key to the therapeutic outcome of cancer by modulating the anti-tumor activity of drugs. Furthermore, the genetic composition of the microbiota and its matching gene sequences in the oncogene has added a new dimension to cancer research. However, it requires adaptive learning techniques and high computational power to bring this research to light empirically. This paper explores the role of machine learning (ML), a subset of artificial intelligence (AI), as a tool to investigate the possible role of the microbiome in the detection and treatment of cancer.

## Introduction and background

Scientific evidence is accumulating for the role played by microbes in human health and disease. An imbalance in the microbiome composition can cause several diseases, including cancer [1]. The microbiota is also thought to affect the therapeutic outcome of cancer by modulating the anti-tumor activity of drugs and influencing the immune system [1]. This indicates the importance of taking the microbiota into consideration, specifically in cancer treatment strategies [2]. In 2017, cancer was estimated as one of the leading causes of death globally, taking the lives of nearly 9.6 million people per year; the Institute for Health Metrics and Evaluation (IHME) provided a lower and upper estimate of 9.4 to 9.7 million deaths [3]. Investigation of the microbiome and understanding its effects on oncogenes may provide additional tools to combat cancer. There is, at the moment, some research happening on this newfound correlation. Findings show that inflammation in the human microbiome is one of the leading causes of cancer formation [1-2,4]. Inflammation can occur for many reasons, such as expansion during pathological dysbiosis that produce high levels of toxins [1-2,4].

Microbiome information on its own may or may not be helpful in prediction, diagnosis, or prognosis. However, it might provide invaluable information in association with other oncogenes and biomarkers status [5]. Research also shows differences in the microbiota composition in cancer patients who respond to treatment and those who do not [5]. This research hints at using the microbiome in precision medicine or individualized medicine in the future [6]. It also shows that many microbes have an anti-tumor effect, while some may have a tumor-promoting effect [1,6]. Lipopolysaccharide, a molecule found in the outer membrane of gram-negative bacteria, and *Lactobacilli, *a genus of gram-positive bacteria,are examples of microbial elements and microbes with anti-tumor effects that activate T-cells to attack cancer cells [1]. On the contrary, certain microbe-associated elements such as *Fusobacterium nucleatum* effector adhesin A (FadA), *Helicobacter pylori *derived cytotoxin-associated gene A (CagA) protein, and *Bacteroides fragilis* metalloproteinase toxin (MP toxin) have tumor-causing effects [1,4]. These molecules and organisms interact with the host cell's epithelial E-cadherins, thereby disrupting the intracellular activities, which in turn causes uncontrollable cell growth and the host cells to transform into cancerous cells [1,4].

Although researchers have identified several beneficial and harmful microbes, there is no complete and extensive list of all the microbes and microbial products that affect cancer development. The problem on hand is that there is limited research done about the correlation between the human microbiome and cancer. Most articles mention the use of metagenomics (study of genetic material) to conduct their research, which lacks effective screening mechanisms for many activities [4]. Thus, the research question remains unanswered: is there a faster, better, and cheaper approach to understand the mysterious link between the human microbiome and cancer? Can science discover a robust solution to save humanity from the deadly disease of cancer? Can advancements in technology, like machine learning (ML), aid in expanding the scope of information processed, reducing time to process such colossal information, and increasing the accuracy of outcomes achieved? Our effort in this paper is to shed some light on the current day techniques used in ML; how artificial intelligence (AI) and ML can be used to identify all the microbes that have a correlation with the development of cancer through oncogenes and, more specifically, match each identified microbe to a specific type of cancer, and showcase other important research that is happening in the field of cancer and microbiome where specific ML techniques were leveraged. Investigation and comparison of microbiome pattern in healthy humans and cancer patients would be necessary. Exploring the effect of microbes on human genes, especially oncogenes, at an epigenetic level (an extra layer of instruction that lies upon DNA and controls how the genes are read and expressed) would then help determine the specific oncogenes affected by each microbe.

## Review

Gut microbiota and cancer

The gut microbiota performs several vital functions like modulating nutrient absorption, vitamin synthesis, protecting against pathogens, immune system development, and epithelial mucosa homeostasis [7,8]. Dysbiosis of the gut, a loss of balance in the equilibrium of microbiota, has been linked to promoting cancer [9]. Bacterial pathogens in a dysbiotic gut can promote cancer by activating inflammation, modulating host immune response, regulating tumor growth and angiogenesis, producing microbial metabolites, and inducing DNA damage [10-14]. Researchers have observed CagA as the first bacterial protein produced by *Helicobacter Pylori* showing involvement in human cancer [15]. They also understood that bacterial pathogens, during infections, may induce DNA instability and breaks of double-strand DNA in gastric mucocytes leading to tumor initiation and growth [16].

On the other hand, studies show that microbiome balance has helped in the prevention of cancer and aided in managing anti-cancer therapies. A high-fiber diet can stimulate gut bacteria to produce short-chain fatty acids such as butyrate that can inhibit the host's tumor cells with an overall anti-cancer effect [17]. Some probiotic-derived molecules and metabolites can modulate the host's immune system, thereby triggering an indirect immune-mediated response against tumor development. For example, a major component of the outer membrane in gram-negative bacteria, bacterial lipopolysaccharide, is said to activate the host's cell surface receptor toll-like receptor 4 (TLR4) [18]. This receptor belongs to the family of pattern recognition receptors (PRRs) and activates immune T cell-mediated response against cancer cells. Many probiotics have shown probable antineoplastic activity [19]. Research indicates that *Lactobacilli *may stimulate the host's immune cells, such as natural killer (NK) cells and dendritic cells (DC), that eliminate cancerous or precancerous cells [20-22]. Many probiotics, such as *Lactobacilli* and Bifidobacteria, can also be used as a supportive treatment for chemotherapy, radiotherapy, and immunotherapy-induced gastrointestinal imbalance: It is due to their ability to restore balance in the gut microbiome [23]. These bacteria can modulate the gut microbiota, strengthen the intestinal epithelium, and protect against inflammation that promotes a pro-cancer environment [24].

While several clinical trials, such as administering probiotics and conducting fecal transplants, have been shown to change the microbiota of patients, it requires careful understanding of the patient's clinical and pathological history [25]. Global efforts to advance microbiome research coupled with technology innovation can open new avenues for developing advanced therapeutics and cancer indicators [26].

Machine learning overview

**Figure 1:**
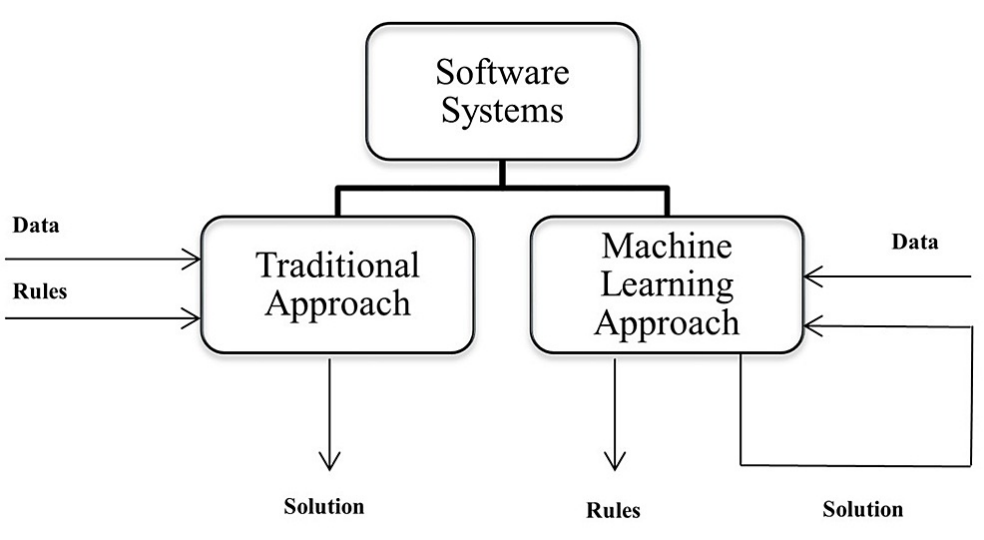
Traditional approach versus machine learning approach

While traditional software systems deal with data and rules to derive a solution, ML, a subset of AI, enables computer systems to automatically learn and improve from experiences without being explicitly programmed (Figure 1). For example, a spam filter program developed using ML techniques is a short and accurate program that automatically and continuously learns which words and phrases could be spam based on spam examples and flags spam content by detecting patterns [27]. ML primarily focuses on developing programs that can access large data sets and learn from them. The learning process begins with capturing observations or collecting data, such as illustrations, direct experiences, or instructions to identify patterns in the data and learn how to make better decisions in the future based on the data feed [27].

The primary goal is to allow the computer systems to learn automatically without human intervention, continuously facilitate intelligent actions, and bring about more and more meaningful outcomes as it evolves. Depending on the type of data used in the research, researchers can use several methods in ML, such as unsupervised learning, supervised learning, and deep learning [28].

Supervised Learning

**Figure 2:**
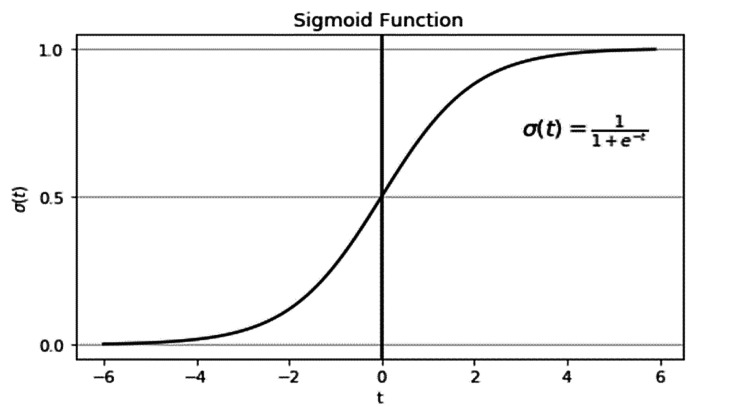
Sample logistic regression (Sigmoid Function)

The system is trained with well-defined input and output parameters (labeled data) in supervised learning. Based on such training, the system now becomes ready to solve complex real-world problems. The commonly used supervised learning methods in the field of microbiome research include:

(i) Random Forest Classifier (RF): This method involves creating and combining multiple decision trees based on the complexity of the problem: The system learns and defines the output based on the majority voting of the individual decision trees [27]. This is the most commonly used technique in patient classification and biomarker analysis [27,28].

(ii) Regression: The output values produced are continuous and based on the training data using a probabilistic interpretation; such regression is further classified as a method called logistic regression (LR) when the output is in between zero and one (Figure 2) [28]. This method is used to solve problems that require easy classification [28,29].

(iii) Support Vector Machines (SVM): This method aims to define what are called support vectors, which are samples closest to a decision boundary between classes [28]. Many problems that require pattern recognition tend to use this method [28,30].

Unsupervised Learning

**Figure 3:**
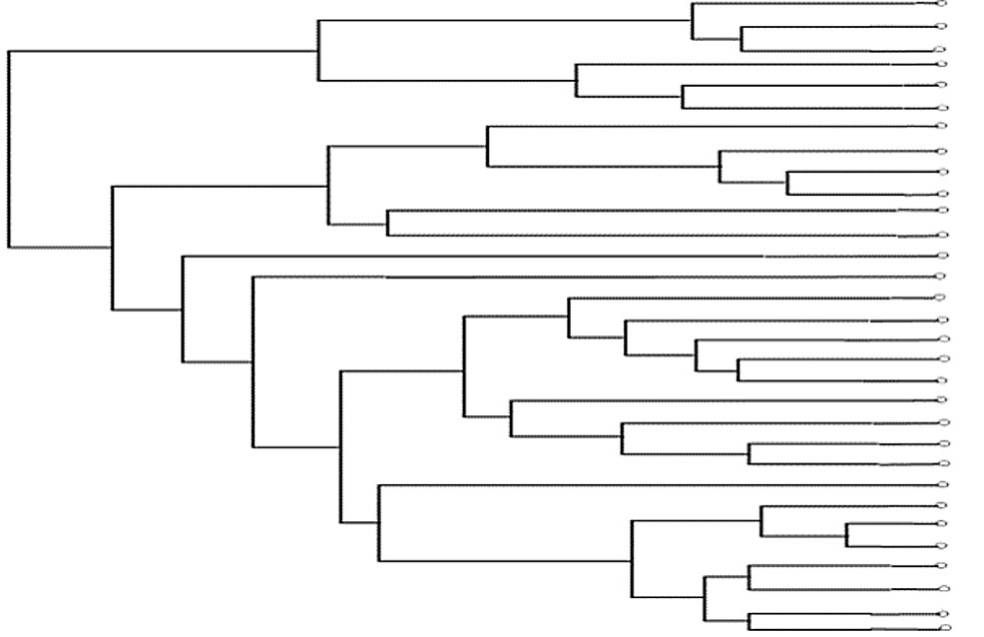
A sample dendrogram cluster

Unsupervised learning is essentially used for processing data with no predefined labels. When researchers want to uncover hidden patterns or decipher information from unstructured data, these techniques come in very handy. Some of such popular techniques include:

(i) Clustering: This method involves creating clusters with groups of similar objects. Hierarchical clustering is one such method that uses a dendrogram to generate nested clusters (Figure 3) [31]. A well-known application of hierarchical clustering is the 16S rDNA sequence analysis used to determine the relationships between various microbes [28,31].

(ii) Dimensionality Reduction: When the data involves too many variables, and interpretation becomes difficult, this method can reduce it into a necessary and meaningful set of dimensions [32].

Deep Learning

**Figure 4:**
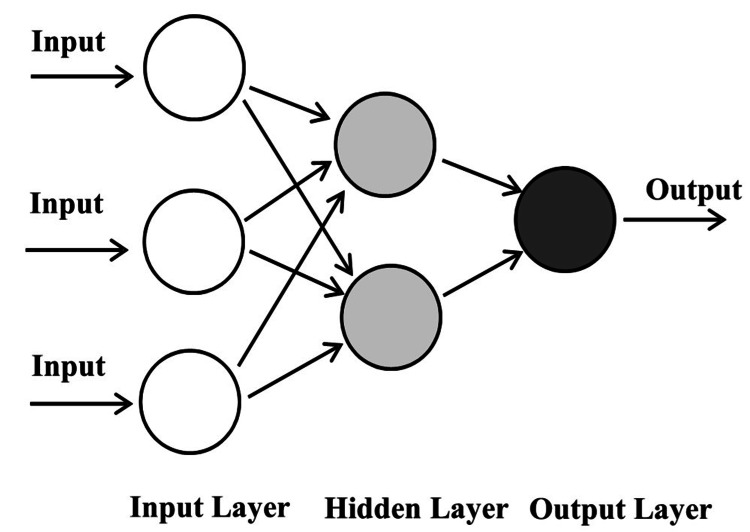
Sample artificial neural network

When the research data becomes large and complex, there may be a need to create a multi-layered network with many hidden layers embedded between the input and output layers. This subset of ML is called deep learning. The most commonly used form of deep learning is artificial neural networks (ANN). This model is based on how the neurons in a human brain operate in a complex multi-layered framework to perform pattern recognition efficiently. While ANNs can be a beneficial tool in the hands of a researcher, the complex layering approach can significantly slow down the data processing speed. Therefore, hardware accelerators like graphics processing units (GPUs) and tensor processing units (TPUs) should replace the conventional central processing unit (CPU) computing power to overcome this limitation [33]. The good news is there are many deep learning frameworks like TensorFlow (open source, developed by the Google Brain team), Pytorch (open source, Facebook's AI Research lab), and MXNet (open source, developed by Apache Software Foundation) that have been highly optimized to process large batches of data and made available as a cloud solution [33]. A sample ANN is shown in Figure 4, and each circle represents a neuron with an activation function. An example of an activation function is a sigmoid function. Each neuron receives a signal, and if the signal is higher than a certain threshold, then the neuron is activated and passes the input to the next layer.

Microbiome and machine learning

Since we will be dealing with a well-defined dataset of microbes and their gene sequences (labeled data), supervised learning techniques like SVM, RF, a combination of SVM and RF (ensemble models), or ANNs are suitable choices for this purpose. These methods depend on the statistical analysis of the data. When boosting algorithms are used in addition, the performance can also be increased. Since a large volume of microbes' genetic data needs to be processed, dimensionality can become an obstacle in getting clean results. For this, many dimensionality reduction techniques can be used to cut down or combine irrelevant features by performing the principal component analysis (PCA) [32].

**Figure 5:**
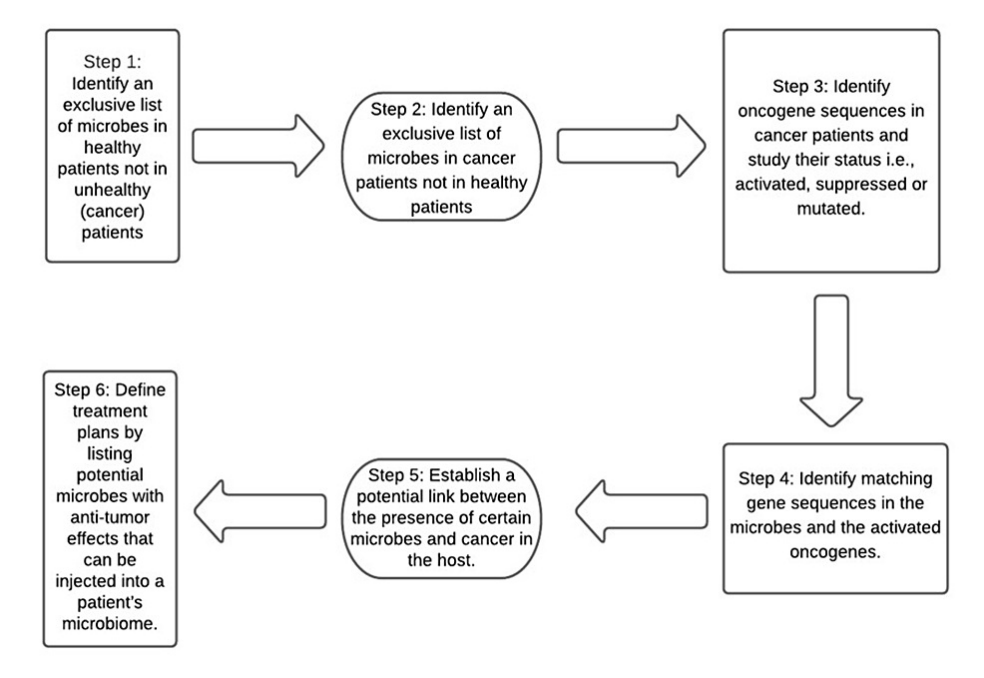
Flowchart showing the steps to identify microbes and their potential tumor or anti-tumor effects.

With the help of one or more of the above ML tools and techniques, it may be possible to establish a link between the identified microbiome and cancer in that host by observing the identified microbes' genetic composition and matching the sequence to the corresponding oncogenes. The very first step is to identify an exclusive list of microbes found in healthy humans and not in unhealthy patients and vice-versa. The microbes found exclusively in healthy humans could potentially have an anti-tumor effect. The microbes solely found in unhealthy (cancer) patients can help expand our research. The second step is to identify the oncogene sequences in the unhealthy patients and study their status, i.e., activated, suppressed, or mutated. The third step is to extract the key features from the gene sequences in the microbes and oncogenes and determine if the oncogenes mutated from the microbes [1,5]. If there is a matching sequence, it may result from the microbe inserting its sequence into the oncogene. In simple terms, ML can apply a filter to the data to keep what is essential for processing. This process will allow us to cut down on the size of the processed data, which will, in turn, lower cost and running time [32]. With all of the extra data cut out, we can see which gene sequences are present in both the microbes and the oncogenes. If their genetic makeups are similar, then we can potentially conclude their association. By completing the above process, we can tell which microbe is causing a certain type of cancer directly or indirectly. On the contrary, if certain microbes are found to have an anti-tumor effect, these microbes can then be injected into a patient's microbiome if they are at risk of developing a certain type of cancer (Figure 5).

Other researchers have used one or more ML techniques in their microbiome research to target cancer detection or identify potential causes or cures. In Table 1 are a few interesting research articles, the corresponding ML tool used, and the primary conclusion derived.

**Table 1:** Microbiome research and machine learning

Research	Machine Learning Technique Used	Primary Conclusion
Flemer et al. [34] Changes in microbiota are connected with colorectal cancer and the prominent presence of putative oral microbes on colonic tumors. This study tried to explore whether colonic mucosa-related taxa are orally inferred if such cases are a particular subset of patients or if the oral microbiome is appropriate for evaluating colorectal cancer growth.	Random Forest	The study showed that it is highly likely that the heterogeneity of colorectal cancer may be connected to microbiota types that are predisposed or provide resistance to the disease, and profiling the oral microbiome may offer an alternative screening mechanism for detecting colorectal cancer.
Koohi-Mugadham et al. [35] This study aimed to discover colorectal cancer-specific metagenomic biomarkers using the application of "MetaMarker", a new approach for discovering metagenomic biomarkers on whole-metagenome sequencing of colorectal cancer stool samples from France.	Random Forest	The study showed the validity of the discovered biomarkers by testing in samples from Hong Kong, Austria, Germany, and Denmark. It proved that these biomarkers could be utilized to develop an Artificial Intelligence classifier for colorectal cancer forecast.
Gupta et al. [36] This study tried to bring about a possible link between the presence of Flavanoid-Degrading Gut Bacterium, Flavonifractor Plautii, and colorectal cancer patients in India.	Random Forest	The study theorized that the degradation of beneficial flavonoids might be playing a role in cancer progression based on research conducted on a certain set of colorectal cancer patients in India. The study also identified 20 potential microbial taxonomic markers and 33 potential microbial gene markers that discriminate the Indian colorectal cancer microbiomes from healthy microbiomes with high accuracy using Machine Learning approaches.
Wirbel et al. [37] This study meta-analyzed fecal metagenomes and uncovered global microbial signatures specific for colorectal cancer. It further established a metabolic link between cancer-associated gut microbes and a fat and meat-rich diet.	Logistic Regression; Random Forest; Clustering	The study confirmed the possible use of globally generalizable, predictive taxonomic, and functional microbiome colorectal cancer signatures as a future diagnostic to detect colorectal cancer.
Cai et al. [31] The study proposed an online learning-based Machine Learning algorithm (ESPIRIT-Tree) to address some of the previously faced efficiency issues in conducting taxonomy independent analysis for microbial community analysis.	Hierarchical Clustering	The study conducted on a human gut microbiota dataset with over one million sequences showed higher effectiveness of a new Machine Learning algorithm (ESPRIT-Tree) which analyzed space and computational complexity in a microbial community analysis that was not addressed in previous algorithms.
Poore et al. [38] This study aims to detect cancer using blood samples and analyze microbial (bacterial and viral) DNA using Machine Learning models.	Stochastic Gradient Boosting Machine Learning Algorithms	The study analyzed microbial patterns in patients' blood samples and identified, using Machine Learning models, individuals with and without any of the three types of cancer and differentiate between the three types of cancer.

Limitations

Although the points discussed earlier in the section alone cannot help create an anti-tumor environment or determine the cause of oncogene development, it can certainly add value in developing efficient ways to find a personalized treatment plan based on the gathered data. However, the challenges to the researcher can be multi-fold. The first challenge is filtering and extracting a consolidated list of microbes unique to cancer patients and unique to healthy humans. These lists can be evolving, and a lack of collaborative approach from the scientific community can become a potential limitation. The next challenge is to extract the key features from the gene sequences in the microbes and oncogenes to determine if the oncogenes mutated from the microbes. However, establishing an association between a microbe and the oncogene does not automatically ensure confirmation. Microbes may indirectly influence or contribute to cancer development by secreting some products like peptides, proteins, or other small molecules. They may cause inflammation, alter a cell's micro-environment, interfere in a cell cycle, or activate or suppress cellular pathways that promote cancer. Researchers who do not account for such indirect factors may end up compromising the accuracy of their predictions. A possible solution for researchers to explore could be to check the genetic sequences of oncogenes for any abnormalities like mutations, deletions, or insertions by matching the reference gene sequence utilizing the National Institutes of Health's GenBank® database. We look forward to seeing how and when the research community can completely overcome such limitations and establish new frontiers in cancer detection and prevention using microbiome data and advanced computational tools of ML.

## Conclusions

Our microbiome study demonstrates an increasing need to process vast amounts of data, including millions of human and bacterial gene sequences, for better outcomes. While this remained a hindrance for decades, the advent of tools like ML opened new frontiers in microbiome research. Ongoing research in oncogene and microbiota is adding tremendous quantities of unstructured data, and turning these big data sets into meaningful ML models is the challenge on hand. The central objective of this research is to stir the interest of the cancer research community in light of new findings in the field of microbiome and bring about a digital transformation to their research strategies to improve the quality of future patient care.
